# An Online Classification Method for Fault Diagnosis of Railway Turnouts

**DOI:** 10.3390/s20164627

**Published:** 2020-08-17

**Authors:** Dongxiu Ou, Yuqing Ji, Rei ZhG, Hu Liu

**Affiliations:** 1 Shanghai Key Laboratory of Rail Infrastructure Durability and System Safety, Shanghai 201804, China; ou.dongxiu@tongji.edu.cn (D.O.); jiyuqing@tongji.edu.cn (Y.J.); 2 School of Transportation Engineering, Tongji University, No.4800 Caoan Road, Shanghai 201804, China; 3 School of Rail Transit, Shanghai Institute of Technology, No.100 Haiquan Road, Shanghai 201418, China; liuhu@sit.edu.cn

**Keywords:** turnout system, fault diagnosis, incremental learning, scalable recognition

## Abstract

Railway turnout system is a key infrastructure to railway safety and efficiency. However, it is prone to failure in the field. Therefore, many railway departments have adopted a monitoring system to monitor the operation status of turnouts. With monitoring data collected, many researchers have proposed different fault-diagnosis methods. However, many of the existing methods cannot realize real-time updating or deal with new fault types. This paper—based on imbalanced data—proposes a Bayes-based online turnout fault-diagnosis method, which realizes incremental learning and scalable fault recognition. First, the basic conceptions of the turnout system are introduced. Next, the feature extraction and processing of the imbalanced monitoring data are introduced. Then, an online diagnosis method based on Bayesian incremental learning and scalable fault recognition is proposed, followed by the experiment with filed data from Guangzhou Railway. The results show that the scalable fault-recognition method can reach an accuracy of 99.11%, and the training time of the Bayesian incremental learning model reduces 29.97% without decreasing the accuracy, which demonstrates the high accuracy, adaptability and efficiency of the proposed model, of great significance for labor-saving, timely maintenance and further, safety and efficiency of railway transportation.

## 1. Introduction

Due to its characteristics of comfort, speed, energy-saving and environmental protection, the high-speed railway has an increasing ratio of carrying passengers and cargo, which puts increasing requirements on the safety of the railway system. According to the 2019 China Railway Statistical Bulletin [1], in 2019, the number of railway passengers sent across the country was 3.66 billion in China, an increase of 8.4% over the previous year. Moreover, in Europe, passenger and cargo transportation in railways has been, respectively doubled and tripled in the past decade [2] according to the report of the European Commission.

The turnout, which is usually installed between two or more strands of tracks, is responsible for switching the train direction, consequently being a key part of the whole signal system in railway infrastructure [3,4,5].

However, due to its complicated mechanical and electrical structure, exposure to the outdoor environment and the need to be frequently pulled, the turnout is more prone to failure. Based on the statistics given by China Railway Jinan Group Co, Ltd. in 2016 [6] and China Railway Guangzhou Group Co, Ltd. in 2018 [7], the number of turnout failures, respectively accounts for approximately 36% and 55% of the total railway signal infrastructure failures. It is obvious that turnout system damage will increase a lot along with the increase in railway transportation demand.

In addition, the cost of turnout maintenance is also very high. For example in England, the maintenance cost of turnout is about 3.4 million pounds per 1000 km of railway [2]. Moreover, according to the report of the International Railway Union, the annual maintenance cost of the turnout system accounts for about 30% of the entire maintenance budget [8]. Moreover, in the United States, the cost is about ten times that of ordinary tracks [9]. Therefore, research on the turnout fault-diagnosis method is very important in not only improving railway reliability and safety, but also in reducing maintenance costs.

The main fault-diagnosis methods have been proposed can be divided into three categories, they are expert system-based, model-based and feature-based.

Xue et al. [10,11,12,13] designed and implemented a fault diagnosis and analysis system for railway signal equipment by establishing an expert knowledge base of railway signal equipment. This method overcomes the overreliance on the mathematical model and can effectively identify the faults. However, the application of expert system needs to obtain sufficient and comprehensive fault diagnosis expert knowledge and experience, which has certain difficulty. In addition, it is also difficult to generalize and summarize the experience of experts into the base [14].

Eker, O. F. et al. [15,16] established the mathematical model for turnouts to achieve fault recognition and prediction of turnout operating state. This method does not require a large number of samples, however, in practice, it is very difficult to establish an accurate mathematical model for complex equipment.

For the feature-based diagnosis method, Witczak, Marcin et al. [17] proposed a turnout fault-diagnosis method based on a neural network. They determined the input parameters and designed a variable threshold to adapt to new faults. Silmon, JA et al. [18] used qualitative trend analysis to extract the data trend of normal and fault samples and established a rule matching mechanism for different fault types.

However, these methods are more costly to deal with new types of faults. Therefore, incremental learning is introduced in this paper. In other engineering areas, some researchers have proposed different classification methods (fault-diagnosis method) based on incremental learning. Generally, these methods can be divided into two different kinds: hierarchy-based methods and improved models [19].

For hierarchy-based methods, a particular learning hierarchy is designed for incremental learning. Nong Ye et al. [20] discussed scalable and incremental learning of a non-hierarchical cluster structure from training data. This cluster structure serves as a function to map the attribute values of new data to the target class of these data, that is, classify new data. Jun Wu [21] proposed a new online semantic classification framework, in which two sets of optimized classification models, local and global, are online trained by sufficiently exploiting both local and global statistic characteristics of videos.

For improved methods, an original model is modified to adapt to the incremental learning scene. Max Kochurov et al. [22] presented an improved Bayesian method to update the posterior approximation of each new data so as to reduce the cost of training a deep learning network. David A [23] proposed an incremental learning target tracking method based on principal component analysis, which effectively adapted to the change of target appearance. Marko Ristin et al. [24] introduced two improved random forest (RF) algorithms, one based on k-means and the other based on SVM, which were used to avoid retraining RFs from scratch when merging new classes. Stefan et al. [25] proposed an incremental learning SVM method, which inputs the data into the algorithm in several batches and produces initial results after each step of training. In addition, many other neural network-based methods have also been proposed [26,27].

Some researchers have also proposed other different incremental methods for classification problems [28,29,30]. However, many of these methods cannot be combined well to the field experience of workers (especially for the turnout system), and also some of these methods cannot update itself, which may reduce the efficiency of the model when new samples or fault types appear since the training samples are quite limited from the very beginning.

Our research group has been long engaged in the safety of railway turnout system, from turnout fault analysis [31] and simulation fault data generating [32], to fault-diagnosis method [33,34,35], based on which, this paper proposed a Bayes-based online turnout fault-diagnosis method with high accuracy, adaptability and efficiency, which can realize incremental learning and scalable fault recognition, i.e., the model can update itself and deal with new turnout fault types, making it more applicable to the fieldwork and eventually, of great significance for labor-saving, timely maintenance and further, safety and efficiency of railway transportation.

**Figure 1:**
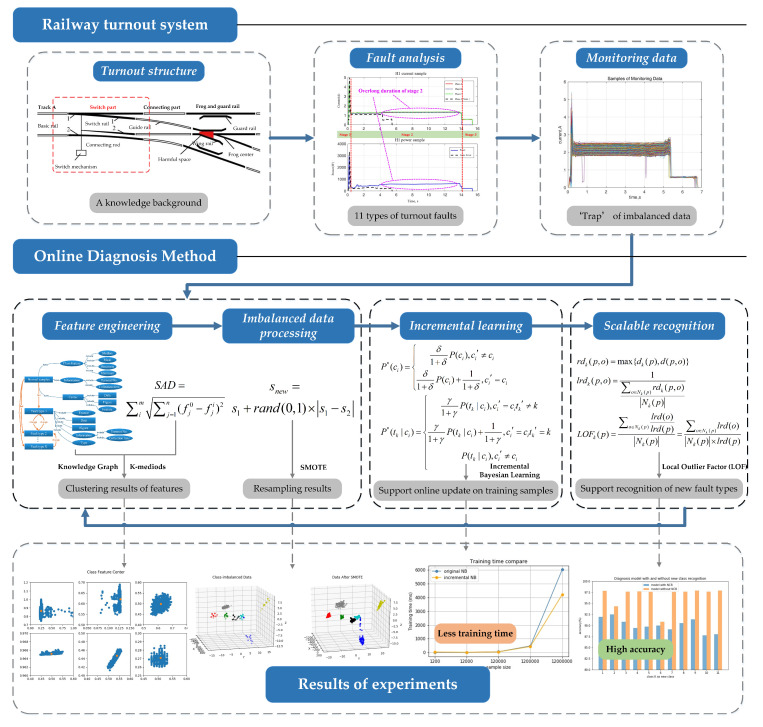
Research framework of this study.

The remainder of this paper is organized as follows: Section 2 introduces the basic conceptions of the turnout system, including its structure, fault types and monitoring data. The methods of data processing and modeling are explained in Section 3, including feature processing, imbalanced data preprocessing, incremental learning method and scalable fault recognition. Section 4 presents a numeric experiment using monitoring data from Guangzhou Railway, followed by conclusions in Section 5. The research framework of this paper is as shown in Figure 1.

## 2. Railway Turnout System

### 2.1. Turnout System

**Figure 2:**
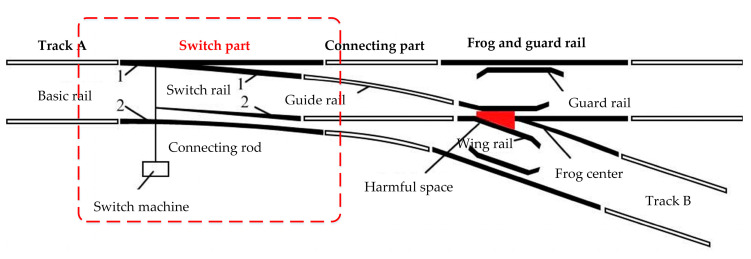
Railway turnout system. The basic structure of a single turnout system and the location of mechanical components.

Turnout is a kind of railway track that can be used to change the direction of the route by moving the rails before the trains pass. The whole turnout system includes an indoor control circuit and an outdoor mechanical structure. Figure 2 [36] shows the basic structure of a single turnout system. As shown in Figure 2, the railway turnout system is mainly composed of three parts, among which, the switch part, where massive monitoring data can be obtained by the MMS (microcomputer monitoring system), plays an important role in switching the train direction.

The switch part, as Figure 2 shows, consists of two basic rails (basic rail 1 and basic rail 2), two switch rails (switch rail 1 and switch rail 2) and a switch machine [37]. Among them, the switch rails are connected with the switch machine through the connecting rod to realize the corresponding operation of the switch machine into horizontal conversion, locking, indication and other operations of the switch rails.

### 2.2. Fault Type Analysis

Due to the complicated mechanical and electrical structure, exposure to the outdoor environment and the need to frequently pull it, the turnout is more prone to failure. According to our and other scholars’ previous research [33,38,39,40] and combined with the field data, the common types of fault in the turnout system are shown in Table 1 [33].

**Figure 3:**
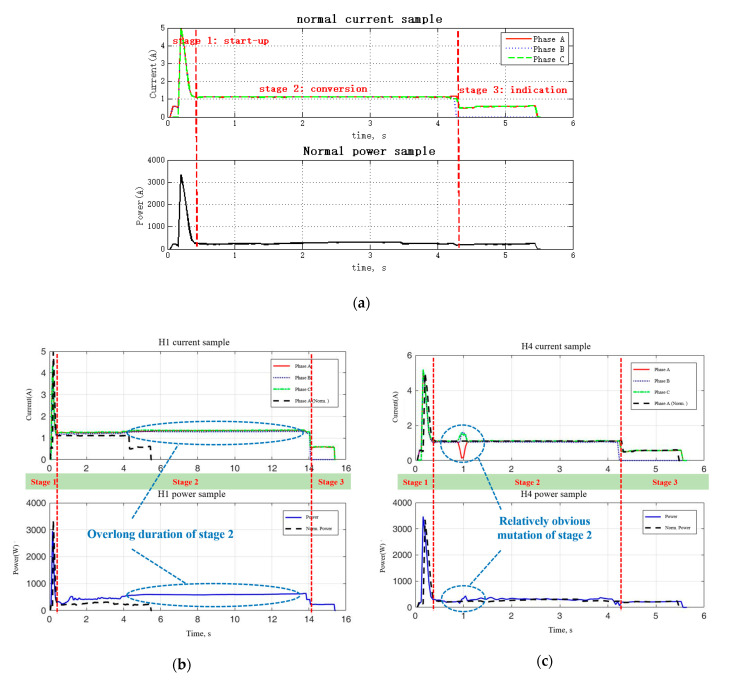

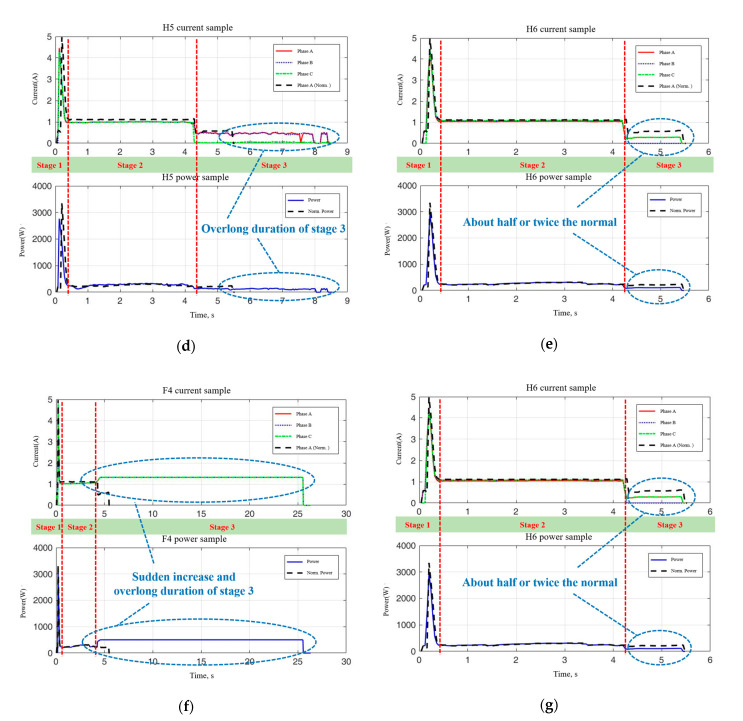
Current and power data of normal and fault types of turnout system. (**a**) Normal sample of current and power curve; (**b**) sample of fault H1; (**c**) sample of fault H4; (**d**) sample of fault H5; (**e**) sample of fault H6; (**f**) sample of fault F4; (**g**) sample of fault F5.

The detailed analysis of monitoring data of the turnout system under normal and abnormal working conditions is as shown in Figure 3. Figure 3a is the normal current and power sample, showing the three stages of a single movement of the turnout, i.e., startup (Stage 1), conversion (Stage 2) and indication (Stage 3).

- Stage 1 is the starting of the switch machine and unlocking of the turnout, which needs to overcome much resistance;
- Stage 2 is the process of the movement of the switch rail to the other basic rail, as well as the locking of the turnout;
- Stage 3 is responsible for indicating the result of the conversion using a low-power indicating circuit.

Figure 3b–g shows the samples of some main fault types, i.e., H1, H4, H5, H6, F4 and F5, which are relatively more frequently occur in the fieldwork. For example, the sample of fault H1 is as shown in Figure 3b, where the duration of Stage 2 is about 14 s, which is too long compared to the normal of about 4 s. As shown in Table 1, this fault is usually caused by mechanical jam, which means there may be a foreign body between the switch rail and the basic rail or there is a jam between the locking gear and the locking block.

### 2.3. Imbalanced Monitoring Data

Railway turnout system is a key infrastructure to railway safety and efficiency; therefore, many railway departments have adopted MMS to monitor turnouts, providing massive data every day. Among all kinds of monitoring data, the current and power data, which are representative and significant indicators of the turnout working condition, are used in this research.

**Figure 4:**
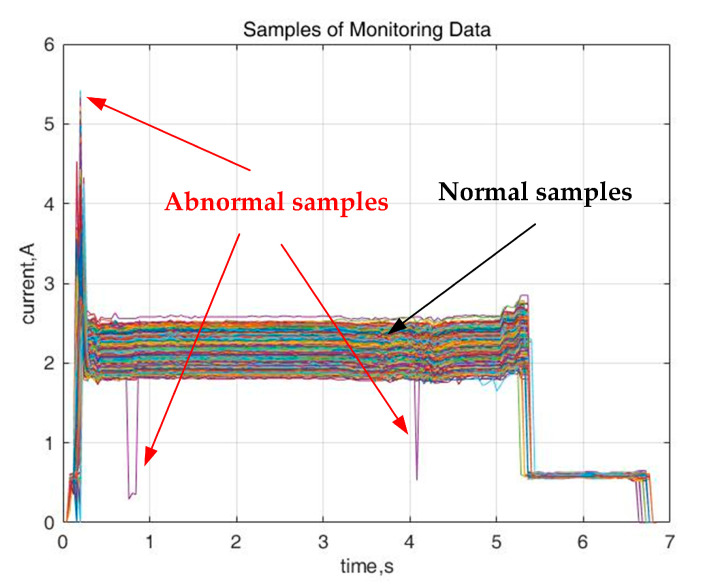
The cumulative current samples from a certain station.

More than 2000 pieces of current samples of a railway station for about five months are shown in Figure 4, where only about 3 abnormal samples can be found, and the rest are normal, which indicates that there is a huge imbalance between the normal and abnormal samples, let alone between the normal and 11 types of fault samples. If provided with these data for training, the classification model is prone to be caught in a ‘trap’ of imbalanced data.

Take an extreme example, suppose there are 1000 samples in total, of which there is only one abnormal sample, then even when the classifier judges all samples as normal, the accuracy can still reach 99.9%, however, missing the most important abnormal sample. Therefore, preprocessing of the imbalanced data are of great significance to improve the performance of the diagnosis model.

## 3. Online Learning and Diagnosis Method

### 3.1. Knowledge Graph-Based Feature Engineering

Feature engineering, which, to a large extent, determines the limit of machine learning, is of great importance to obtaining valuable input features for the online diagnosis model of this paper. Therefore, the first step is feature extraction from the original data. The feature extraction method for monitoring data of railway turnouts has been introduced in our previous research [33], where 21 features (e.g., maximum value, mean value, fluctuation factor, peak factor, etc.) are stored by the knowledge graph in this paper.

**Figure 5:**
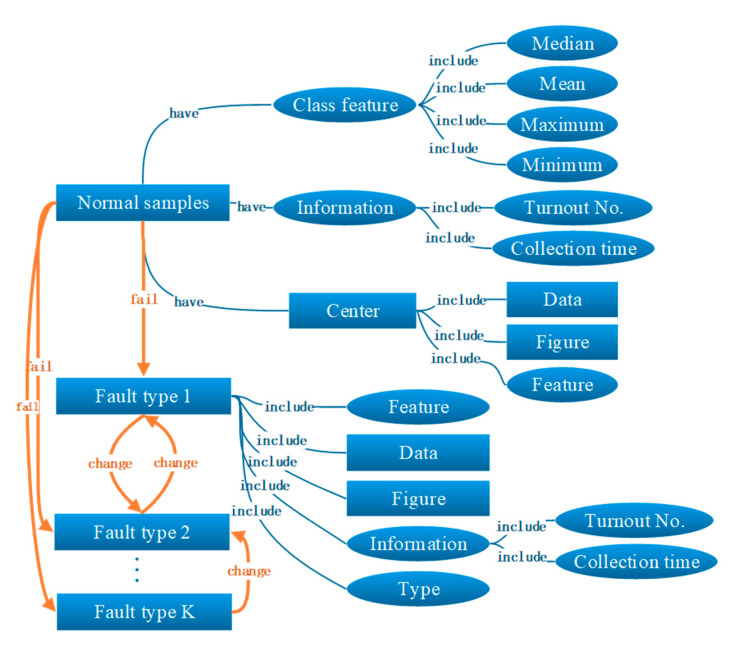
Structure of feature-based knowledge graph.

The knowledge graph is a theoretical method to visualize the core structure or knowledge framework of disciplines or research fields, where the representation of the knowledge graph may vary from one another. In this paper, the entity-relationship model is used to show the structure of the knowledge graph of the turnout monitoring data, where the basic elements are entities (squares), attributes (ellipses) and relationships (lines) as shown in Figure 5.

In reality, turnout systems are well maintained, and the fault samples are quite rare. According to the data from Guangzhou Railway, the abnormal samples account for less than 0.1% of the total. Therefore, the knowledge graph of normal samples and abnormal samples should be arranged in different ways.

As shown in Figure 5, for normal conditions, it is not necessary to put every single sample into the knowledge graph. Instead, the changing trend of sample features is the most important characteristic. Therefore, the knowledge graph records the center, i.e., the most representative sample of all the normal samples. Other normal samples are only recorded as feature ranges, e.g., mean, maximum, etc. For abnormal conditions, the samples are divided into different fault types. In each fault type, all samples will be recorded into the knowledge graph, including features, figures, data and information.

As mentioned above, it is very important to choose the center of normal samples and record it into the knowledge graph. After the feature set is formed, the next step is to choose the most representative sample, i.e., the center. To solve this problem, the K-medoids method (an adaption of K-means algorithm) [35], which requires the clustering center to be one of all the samples rather than creating a new clustering center, is applied in this paper. The steps of the K-medoids algorithm are as follows:

- The input feature set is $F_{m\times n}$ (*n* is the feature dimension) and choose any row as the original center;
- Define the cluster evaluation variable sum of absolute differences (*SAD*):
  (1) $$SAD=\sum_{i}^{m}\sqrt{\sum_{j=1}^{n}{\left(f_{j}^{0}-f_{j}^{i}\right)}^{2}}$$
- Choose any sample except $F^{0}$ as $F^{k}$ and calculate the corresponding *SAD*;
- If the *SAD* of $F^{k}$ is smaller than the *SAD* of $F^{0}$, update the $F^{k}$ to be the new center;
- When all the samples have been searched, the *SAD* of the current center will reach the minimum and the iteration is finished. Otherwise, return to the third step).

### 3.2. Imbalanced Data Preprocessing

Before incremental learning modeling, the imbalanced problem of the dataset needs to be solved. In reality, since fault samples are quite rare, which may influence the classification accuracy. The class imbalance problem means that there is a class in the data set whose sample number is far more or less than others, which often leads to the failure of some machine learning models. In this study, the SMOTE (synthetic minority oversampling technique) [41], an improved method based on random oversampling, is applied to deal with the imbalance data.

SMOTE produces new samples for the minority class by selecting k-nearest neighbors and add random numbers to them. The detailed process is as follows:

- For each sample $s_{1}$ in the minority class, the Euclidean distance is taken as the standard to calculate its distance to all samples in the class so as to find its k-nearest neighbor;
- A sampling ratio N is set according to the sample imbalance ratio; For each minority sample $s_{1}$, several samples are randomly selected from its k-nearest neighbors, assuming that the chosen nearest neighbor is $s_{2}$;
- For each chosen neighbor $s_{2}$, a new sample $s_{new}$ is constructed according to the following formula with the original sample $s_{1}$:
  (2) $$s_{new}=s_{1}+rand(0,1)\times \left|s_{1}-s_{2}\right|$$

### 3.3. Bayesian Incremental Learning

The naïve Bayesian (NB) classification model is a pattern recognition method based on the Bayesian principle which combines the class prior probability and the conditional probability of each feature attribute to calculate the posterior probability.

Presume that the feature set T consists of $\left\{t_{1},t_{2},\ldots ,t_{m}\right\}$ and the m feature attributes are independent of each other. The class variable is represented as $\left(c_{1},c_{2},\ldots ,c_{n}\right)$. A single sample is represented as <T,c>.

According to the Bayesian principle, when the prior probability $P(c_{i})$, the conditional probability $P(T|c_{i})$ and marginal probability is known, the posterior probability $P(c_{1}|T),\;P(c_{2}|T),\;\ldots ,\;P(c_{n}|T)$ corresponding to n classes can be calculated. The largest posterior probability indicates the class of this sample:

(3) $$P(c_{i}|T)=\frac{P(c_{i})\times P(T|c_{i})}{P(T)}$$

The Laplace estimation is applied to get the prior probability and conditional probability of each feature attribute:

(4) $$P(c_{i})=\frac{1+\left|D_{c_{i}}\right|}{\left|C\right|+\left|D\right|}$$

(5) $$P(t_{k}|c_{i})=\frac{1+\left|D_{t_{k}|c_{i}}\right|}{\left|B_{k}\right|+\left|D_{c_{i}}\right|}$$

where $\left|D_{c_{i}}\right|$ is the number of the sample of class $c_{i}$ in the training data, |C| denotes the class number, |D| denotes the number of training samples, $\left|D_{t_{k}|c_{i}}\right|$ is the number of training samples which have the attribute $t_{k}$ and belongs to the class $c_{i}$ and $\left|B_{k}\right|$ is the number of training samples which have the feature attribute k. Since the features are independent of each other, the conditional probability can be calculated as:

(6) $$P(T|c_{i})=\prod_{k=1}^{m}P(t_{k}|c_{i})$$

naïve Bayesian classifier is of strong mathematical logic and high classification accuracy. However, the traditional naïve Bayesian classifier has two weaknesses. First, the classification accuracy of the classifier is closely related to the size and integrity of the training data set, which, generally, is difficult to guarantee. Second, when it is applied to turnout fault diagnosis, with the sample size gradually increasing, for each additional batch of sample data, the quasi-prior probability and conditional probability of each characteristic attribute must be recalculated, which is very time consuming and cannot meet the real-time requirement of online fault diagnosis.

However, the combination of the Bayesian algorithm and incremental learning can solve these problems well while maintaining its inherent strong logic and high diagnostic accuracy. When a fault occurs, the newly added sample is input into the Bayesian classification model to get the diagnosis result. If the result is reliable, then this new sample can be used to update the model and ultimately helps to increase the sample data sets and accuracy of the model.

For the new sample, when meeting the judgment conditions, it can be used to update the model, i.e., to update the class prior probability and characteristic properties of conditional probability, which are defined as follows:

(7) $$P^{*}(c_{i})=\{\begin{array}{c} \frac{\delta }{1+\delta }P(c_{i}),c_{i}{}^{\prime }\neq c_{i}\\ \frac{\delta }{1+\delta }P(c_{i})+\frac{1}{1+\delta },c_{i}{}^{\prime }=c_{i} \end{array}$$

(8) $$P^{*}(t_{k}|c_{i})=\{\begin{array}{c} \frac{\gamma }{1+\gamma }P(t_{k}|c_{i}),c_{i}{}^{\prime }=c_{i}t_{k}{}^{\prime }\neq k\\ \frac{\gamma }{1+\gamma }P(t_{k}|c_{i})+\frac{1}{1+\gamma },c_{i}{}^{\prime }=c_{i}t_{k}{}^{\prime }=k\\ P(t_{k}|c_{i}),c_{i}{}^{\prime }\neq c_{i} \end{array}$$

where $P^{*}(c_{i})$ is the updated class prior probability and $P^{*}(t_{k}|c_{i})$ is the updated conditional probability. To be specific, δ=|C|+|D| and $\gamma =\left|B_{k}\right|+\left|D_{c_{i}}\right|$.

As for the judgment conditions, the posterior probability of the fault label $c_{i}{}^{\prime }$ satisfies the following condition:

(9) $$P(c_{i}{}^{\prime }|T)\geq \alpha (1-P(c_{i}{}^{\prime }|T))$$

**Figure 6:**
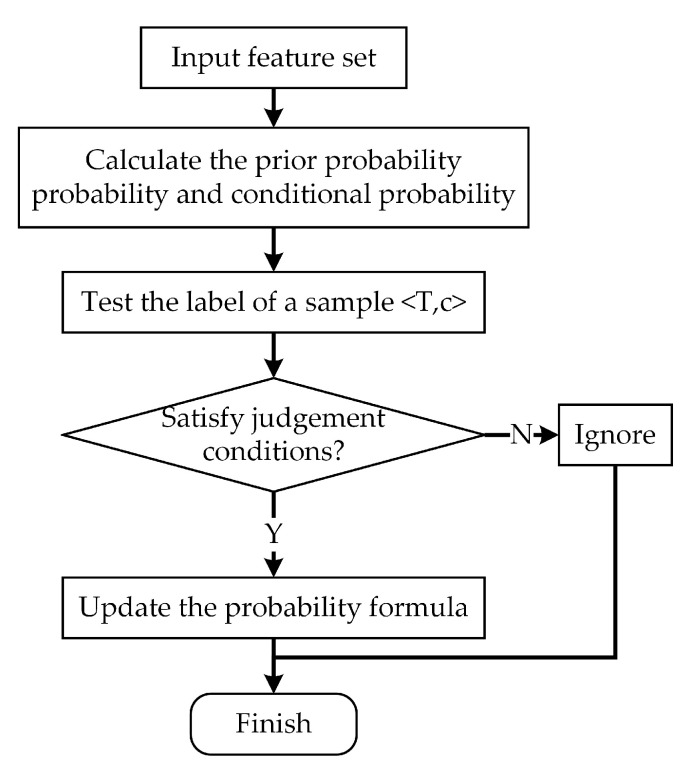
Flow chart of Bayesian incremental learning.

The overall incremental fault diagnosis process is as shown in Figure 6.

### 3.4. Scalable Fault Recognition

Though the incremental learning method was widely used in different fields and it can reduce the training cost, there is a key problem that cannot be ignored is the recognition of new classes. Since the types of faults may increase along with the mechanical changes of the turnouts, the new fault types are very normal in reality. Therefore, to find a method to recognize new faults and realize the automatic update of the knowledge graph is very important for the field application.

The scalable fault recognition problem (SFR) can be abstracted into a novelty detection problem. The model of novelty detection will fit a rough boundary to define the contour of the initial samples, i.e., the normal samples. When a new sample appears, it will be judged whether it is within the boundary.

Local outlier factor (LOF) [42], an efficient novelty detection method, aims to detect those abnormal data that are quite different from the characteristic attributes of normal data. One of the assumptions of LOF is that the normal data must be clustered, which puts forward certain requirements on the original data set. The turnout data samples, which were clustered according to the mentioned methods of data processing, basically meet this requirement.

The principle of LOF is to calculate the density of a certain point and to judge whether the point is in the sparse region according to the density of the data cluster of the dataset itself. A point in the sparse region is recognized as a novelty point. Due to its "local" characteristics, LOF can handle clusters with different densities correctly. The local outlier factor $LOF_{k}(p)$ is used to measure the novelty degree of point p:

(10) $$rd_{k}(p,o)=\max\{d_{k}(p),d(p,o)\},$$

(11) $$lrd_{k}(p,o)=\frac{1}{\frac{\sum_{o\in N_{k}(p)}rd_{k}(p,o)}{\left|N_{k}(p)\right|}},$$

(12) $$LOF_{k}(p)=\frac{\sum_{o\in N_{k}(p)}\frac{lrd(o)}{lrd(p)}}{\left|N_{k}(p)\right|}=\frac{\sum_{o\in N_{k}(p)}lrd(o)}{\left|N_{k}(p)\right|\times lrd(p)},$$

where d(p,o) is the distance between point p and o;

$d_{k}(p)$ is the distance between point p and the $k_{th}$ nearest neighbor of p;

$rd_{k}(p,o)$ is the reachability distance between point p and o;

$lrd_{k}(p,o)$ is the local reachability density. It measures the density of point p and its k neighbors. The larger the reachable distance, the smaller the density;

$LOF_{k}(p)$ is the local outlier factor. It defines a concept of relative density, which can deal with heterogeneous data and make outlier detection more accurate.

According to the definition of the local outlier factor, $LOF_{k}(p)\approx 1$ means the local density of p is similar to its k neighbors; $LOF_{k}(p)$ < 1 means p is in a high-density area, indicating it is a normal point; If $LOF_{k}(p)\gg 1$, then the point p is far away from the normal data cluster, which means it is very likely to a novelty point.

Since the original LOF can only deal with binary classification problems, the final output should consider all the binary classification models, inspired by the multiple classification models. If all the binary classification models output −1, i.e., this sample is not similar to any known classes, then it is identified as a new class sample.

When the sample size is big enough, a clustering method is applied, and the unidentified samples can be labeled more easily. Finally, the densest N clusters among these unidentified samples are selected as the candidate sample set of the new classes. If the density of this sample set exceeds the specified threshold, then it is determined as the representative sample set of the new class for further knowledge graph update.

## 4. Experiment Result

### 4.1. Clustering and Resampling

**Figure 7:**
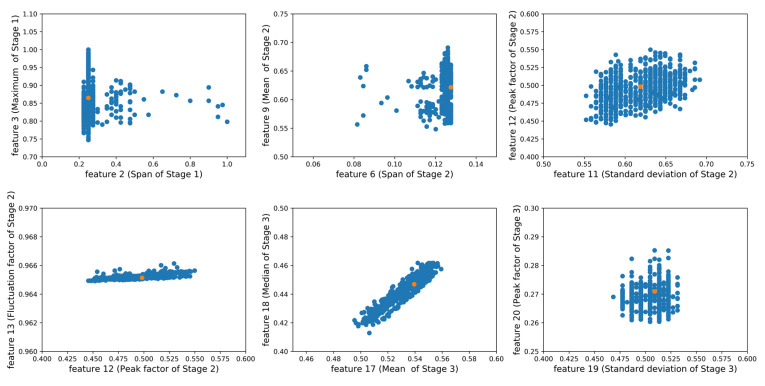
Class center of features.

In this paper, the experimental data are from several stations of Guangzhou Railway from 2016 to 2018. As mentioned before, the class center is the most representative sample of a class and one of the key components of the knowledge graph. Taking the normal class as an example, the dimension of features is 21, under which, the feature clustering result is abstract and hard to understand. For a clearer display, the clustering results are divided into several pairs, e.g., feature 2 (span of Stage 1) & feature 3 (maximum of Stage 1), feature 6 (span of Stage 2) & feature 9 (mean of Stage 2), feature 11 (standard deviation of Stage 2) & feature 12 (peak factor of Stage 2), etc., as shown in Figure 7, where the orange point stands for the center, and the values of all features have been normalized.

Before using the feature set to train the diagnosis model, the class-imbalanced data should be preprocessed with the SMOTE resampling method. In this paper, the original data set consists of 1000 normal samples and 10 samples of each 11 fault types, and the final data set includes 12,000 samples in total (each class with 1000 samples).

**Figure 8:**
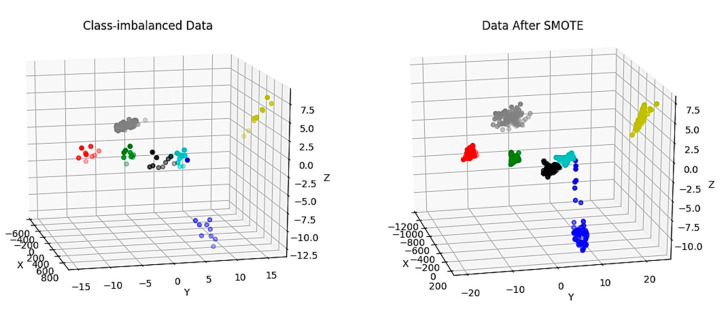
Date of original and resampled.

To show it more clearly, an example data set is organized with 100 normal samples, and each of 6 different fault types is of 10 samples, i.e., the normal sample size to the abnormal is 10:1. Moreover, an LDA feature reduction method is applied to reduce the feature dimension to 3, which can be easily shown in the picture. Figure 8 shows the original example data set and the resampled data set after SMOTE. The fault samples are multiplied according to the original imbalance ratio. After SMOTE, the sample sizes of all classes (including the normal class) are the same. This method cannot only be used in data preprocessing, but also in generating fault data manually.

### 4.2. Incremental and Scalable Fault Diagnosis Model

With the input data set prepared, it comes to the next step, the scalable fault recognition. Different parameters can be set to get the scalable fault recognition accuracy of different models to determine the optimal parameter value. The main parameters of the LOF model are “n_neighbors” (n), which is the number of neighbors and “contamination” (c), the percentage of outliers in the test sample. With the method of grid search, the parameter choosing result is shown in Table 2. Therefore, the optimal values for the parameters should be n = 40 and c = 0.01.

**Figure 9:**
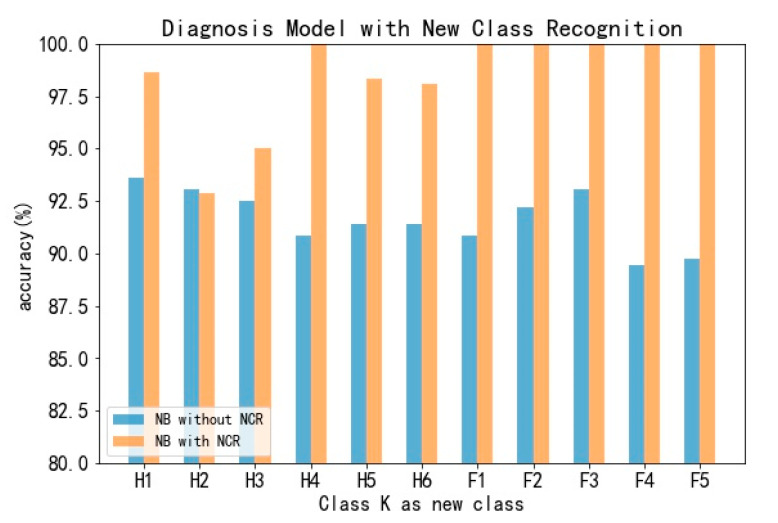
Result of scalable fault recognition.

For the experiment, only the samples not identified as a new class can be input into the diagnosis model. For a single fault type, which is regarded as a new class, the diagnosis models with and without scalable fault recognition are compared. As shown in Figure 9, the blue bars represent the accuracy of the original model and the yellow bars represent the accuracy of the model with scalable fault recognition. To 11 fault types, the accuracies improve to different degrees and the average accuracy improves about 6.8%. Moreover, the total accuracy of scalable fault recognition is 99.11%. When the training data increasing over time, the accuracy will increase as well.

**Figure 10:**
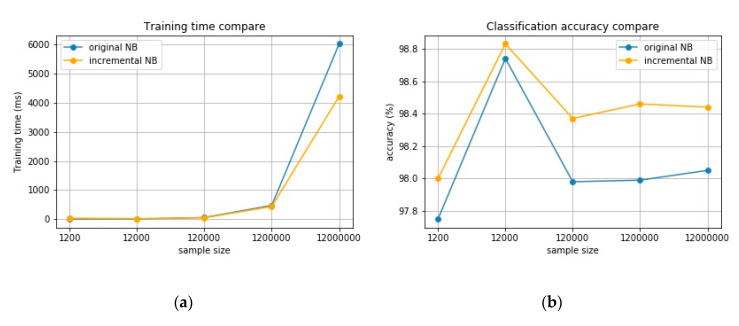
Comparison of original naïve Bayesian (NB) and incremental NB. (**a**) Comparison of training time; (**b**) comparison of classification accuracy.

After leaving out the new class samples, the incremental Bayesian model and naïve Bayesian model are compared both in training time and accuracy. The incremental NB model divides the whole data set into 10 batches and trains them separately. As shown in Table 3 and Figure 10a, it can be easily found that as the sample size increases, the incremental NB takes less training time than the original NB. When the sample size reaches 12 million, the training time of incremental NB decreases by about 30%. For the training time, the larger the training data set is, the greater the gap between the two models will be. At the same time, it can be found in Table 4 and Figure 10b that the accuracy of incremental NB is always above the original NB, with an average improvement of 0.32%. This is mostly caused by the step-by-step training model of incremental NB, which can choose the most valuable samples for training.

## 5. Conclusions

Based on imbalanced monitoring data, this paper proposes a solution for railway turnout fault diagnosis, which can realize incremental learning and scalable fault recognition. First, this paper introduced the basic conceptions of the railway turnout system. Next are the knowledge graph-based feature engineering and processing of the imbalanced monitoring data. Then, an online learning and diagnosis method based on Bayesian incremental learning and scalable fault recognition is proposed. According to the results of the experiment, with data from Guangzhou Railway, the scalable recognition method can reach an accuracy of over 99%. Moreover, compared to the non-incremental model, the incremental learning model saves about 30% of training time when the sample size reaches 12 million—with a slight accuracy improvement as well. In addition, it should be noted that the proposed solution is applicable to most railway turnout systems, except for those whose switch machines are of three-phase AC type.

Future research directions of this paper may include studying a more intelligent method to improve the accuracy of scalable fault recognition, and further improvement of the knowledge graph structure is also required.

## Figures and Tables

**Table 1:** Turnout fault types.

Fault Label	Fault Type
H1 ^1^	Mechanical jam
H2	Improper position of the slide chair
H3	Abnormal impedance in the switch circuit
H4	Bad contact in the switch circuit
H5	Abnormal open-phase protection device in the indicating circuit
H6	Abnormal impedance in the indicating circuit
F1 ^2^	The electric relay in the start circuit fails to switch
F2	Supply interruption
F3	Open-phase protection device fault
F4	Fail to lock
F5	Indicating rod block in the gap

^1^ H—hidden dangers, showing that the turnout system is in an unhealthy situation, which may further cause major faults; ^2^ F—major faults that are more serious.

**Table 2:** Local outlier factor (LOF) parameter choosing result (MS).

	**n**	25	30	35	40
**c**					
0.0001		95.35	95.35	93.76	98.13
0.001		95.35	95.35	93.76	98.13
0.01		95.38	95.83	96.01	99.11

**Table 3:** Training time comparison (MS).

**Sample size**	1200	12,000	120,000	1,200,000
**NB**	6.62	10.27	59.84	476.52
**Incremental NB**	38.48	19.53	51.32	436.72

**Table 4:** Classification accuracy comparison (%).

**Sample size**	1200	12,000	120,000	1,200,000
**NB**	97.75	98.74	97.98	97.99
**Incremental NB**	98.00	98.83	98.37	98.46
